# Does the interval from tumour surgery to radiotherapy influence survival in paediatric high grade glioma?

**DOI:** 10.1007/s00066-018-1260-z

**Published:** 2018-01-18

**Authors:** Amedeo A. Azizi, Simon Paur, Alexandra Kaider, Karin Dieckmann, Andreas Peyrl, Monika Chocholous, Thomas Czech, Irene Slavc

**Affiliations:** 1 Department of Pediatrics and Adolescent Medicine, Medical University of Vienna, Spitalgasse 23, 1090 Vienna, Austria; 2 Center for Medical Statistics, Informatics, and Intelligent Systems, Medical University of Vienna, Vienna, Austria; 3 Department of Radiation Oncology, Medical University of Vienna, Vienna, Austria; 4 Department of Neurosurgery, Medical University of Vienna, Vienna, Austria

**Keywords:** Radiotherapy, High grade glioma, HGG, Children, Paediatric oncology, Strahlentherapie, Radiotherapie, Hochgradiges Gliom, HGG, Kinder, Pädiatrische Onkologie

## Abstract

**Purpose:**

Paediatric high grade glioma (pHGG) are rare. Following maximum safe resection, children >3 years with HGG receive radiotherapy as standard of care. Whether the interval from tumour surgery to radiotherapy (ISRT) influences survival is disputed in adults with glioblastoma, data for children are lacking. This retrospective single-centre analysis investigates a possible impact of ISRT on survival in paediatric patients with HGG.

**Methods:**

Survival was analysed in patients aged 3–19 years with non-pontine HGG.

**Results:**

Thirty-eight patients were included (female:male 19:19) with a median age of 11.0 years (3.4–17.7). Seventeen patients had grade 3 and 21 grade 4 glioma. Gross total resection was achieved in 26.3%, partial resection in 36.8% and 36.8% underwent biopsy only. All patients received concomitant and adjuvant chemotherapy. Fifty percent (*n* = 19) started irradiation ≤17 days (median interval 12 days [range 5–17]), 50% thereafter (median 28 days [range 19–78]). More patients with grade 4 tumours were irradiated shortly after surgery. ISRT (as a continuous variable and dichotomised into two groups by the median ISRT of 18 days) did not significantly influence overall survival (OS) or progression-free survival (PFS). Higher extent of resection (EOR), lower tumour grade as well as chemotherapy with temozolomide had a significant positive impact on OS and PFS in univariate analysis and (except for the effect of temozolomide on PFS) also in multivariable analysis.

**Conclusions:**

ISRT did not influence survival in pHGG. In view of upcoming targeted treatment options in pHGG the present data suggest that it is safe to perform molecular analyses within a 4-week timeframe before radiotherapy.

## Zusammenfassung

**Zielsetzung:**

Hochgradige Gliome bei Kindern (pHGG) sind selten. Als Therapiestandard gilt die Tumorresektion gefolgt von Strahlentherapie. Ein möglicher Einfluss des zeitlichen Intervalls zwischen Tumorresektion und Radiotherapie (ISRT) auf das Überleben wird bei Erwachsenen kontroversiell diskutiert. Daten bei Kindern fehlen bis dato. Diese retrospektive unizentrische Studie analysiert das ISRT und dessen möglichen Einfluss auf das Überleben bei pädiatrischen HGG-Patienten.

**Methoden:**

Bei Patienten im Alter zwischen 3–19 Jahren mit nichtpontinem HGG wurde eine Überlebensanalyse durchgeführt.

**Ergebnisse:**

In die Studie konnten 38 Patienten eingeschlossen werden (weiblich:männlich 19:19; medianes Alter 11,0 Jahre [Spanne 3,4–17,7]). 17 Patienten hatten ein Gliom WHO Grad 3 und 21 Patienten vom Grad 4. Eine Totalresektion erhielten 26,3 %, eine Teilresektion bzw. eine Biopsie je 36,8 %. Alle Patienten bekamen eine konkomitante und adjuvante Chemotherapie. Jeweils 50 % (*n* = 19) begannen die Bestrahlung ≤17 Tage (medianes Intervall 12 Tage [Spanne 5–17]), 50 % danach (Median 28 Tage [Spanne 19–78]). Mehr Patienten mit WHO-Grad-4-Tumoren wurden knapp nach der Operation bestrahlt. ISRT (als kontinuierliche Variable und dichotomisiert in zwei Gruppen mittels medianem ISRT von 18 Tagen) hatte keinen Einfluss auf Gesamtüberleben (OS) oder progressionsfreies Überleben (PFS). Ein größeres Ausmaß der Resektion, niedrigerer Tumorgrad und Chemotherapie mit Temozolomid hatten einen signifikant positiven Einfluss auf OS und PFS in univariater und auch in multivariabler Analyse (außer für den Effekt von Temozolomid auf das PFS).

**Schlussfolgerung:**

ISRT hat keinen Einfluss auf das Überleben bei pHGG. Angesichts neuer gezielter Behandlungsansätze bei pHGG erscheint aufgrund der gegenwärtigen Daten eine Frist von 4 Wochen als sicher, um eine molekulare Analyse vor Beginn der Strahlentherapie durchzuführen.

## Introduction

Prognosis of high grade glioma (HGG) in childhood is similarly poor as in adults. Standard of care in children with HGG older than three years of age includes maximal safe resection followed by focal radiotherapy (RTX) and variable adjuvant chemotherapy (CTX). Because HGGs are fast growing, aggressive malignancies neuro-oncology practice usually aims at minimising delay to initiation of RTX after surgery. More recently though, several distinct epigenetic and biological subgroups of HGG gliomas in children were described requiring determination of molecular markers for treatment stratification in future trials [1], thus most likely leading to longer delays in initiation of treatment. Yet no data exist in the paediatric HGG population whether the interval from surgery to initiation of radiotherapy (ISRT) influences survival. From a biological point of view and considering growth dynamics of malignant solid tumours such as high grade glioma an increased risk of relapse and a subsequent negative impact on survival may be postulated [1]. In adults delayed initiation of RTX was indeed proven to negatively impact outcome in head and neck, breast and other cancers [2]. In recent years an increasing number of studies investigated ISRT in adult patients with HGG, but results remain conflicting. Whereas some studies could identify the expected negative impact on survival [3–7], others failed to show any correlation with survival [8–12]. Some large studies even suggest a positive effect of moderately delayed RTX on survival in adult patients with HGG [13–18].

The present institutional study investigates whether the interval between surgery and initiation of RTX influences survival in paediatric patients with HGG.

## Methods

Patients with HGG treated at the Department of Paediatrics, Medical University of Vienna, Austria, from 1995 to 2015 were included in this retrospective single-centre analysis. The following eligibility criteria had to be met: histologically confirmed high-grade glioma (WHO grades 3 or 4), age 3–19 years at diagnosis, application of postoperative adjuvant radiation therapy and sufficient clinical data on primary treatment and follow-up. Patients with diffuse intrinsic brainstem glioma (DIPG) were excluded, as well as patients with a secondary tumour following prior cranial RTX. Patients younger than three years were excluded as RTX is generally withheld in these children [19]. Follow-up was conducted according to institutional standards (Table 1) and was continued at the Department of Paediatrics even after patients had reached adulthood.

**Table 1:** Institutional follow-up for patients with paediatric high grade glioma

Clinical and neurological examination	At least every month during therapy
	Every 3 months for 2 years following therapy
	Then every 6 months
Local MRI (i. e. brain or spine)	Every 3 months during therapy
	Every 3 months for 2 years following therapy
	Then every 6 months
	At clinical progression
Neuro-axis MRI	In case of metastatic disease
	Symptoms suggestive for dissemination
Endocrine surveillance	At baseline
	Every 6 to 12 months following radiotherapy
Hearing	Every 12 months
Neuropsychological testing and support	Routinely during therapy and follow-up
Other (e. g. ophthalmology, bone age, orthopaedics, dermatology)	As clinically indicated

*MRI* magnetic resonance imaging

This retrospective analysis was approved by the ethical review board of the Medical University of Vienna.

### Statistical analysis

Statistical calculations and graphical representation of results was performed using SAS (version 9.4, SAS Institute Inc. [2002–2012], Cary, NC, USA). Comparisons between patient groups with respect to categorical variables were performed using the Chi-square test. The median follow-up times were estimated by the inverse Kaplan–Meier method [20]. Probabilities of progression-free survival (PFS) and overall survival (OS) were calculated by the Kaplan–Meier method and differences in the survival probabilities between patient groups were tested by the log-rank test [21]. Univariate and multivariable Cox regression models [22] were used to evaluate the unadjusted and adjusted effects of the ISRT (in days), the extent of resection (EOR, ranging from biopsy (1) to gross total (4)) and WHO grading (4 vs. 3) on PFS and OS, respectively. Log2-transformed values of ISRT were used for statistical analyses because of their skewed distribution. Additional exploratory analyses were performed, evaluating ISRT and EOR as binary variables, categorizing ISRT according to the median value (≥18 days vs. ≤17 days) and EOR comparing gross total resection to all other resections combined including biopsy. Furthermore, age (in years) was evaluated in univariate regression models as potential prognostic factor for OS and PFS. The Firth correction [23] was used in all Cox regression analyses to reduce bias in the estimates resulting from the rather small sample size. Two-sided *p*-values <0.05 were considered as indicating statistical significance.

## Results

**Fig. 1:**
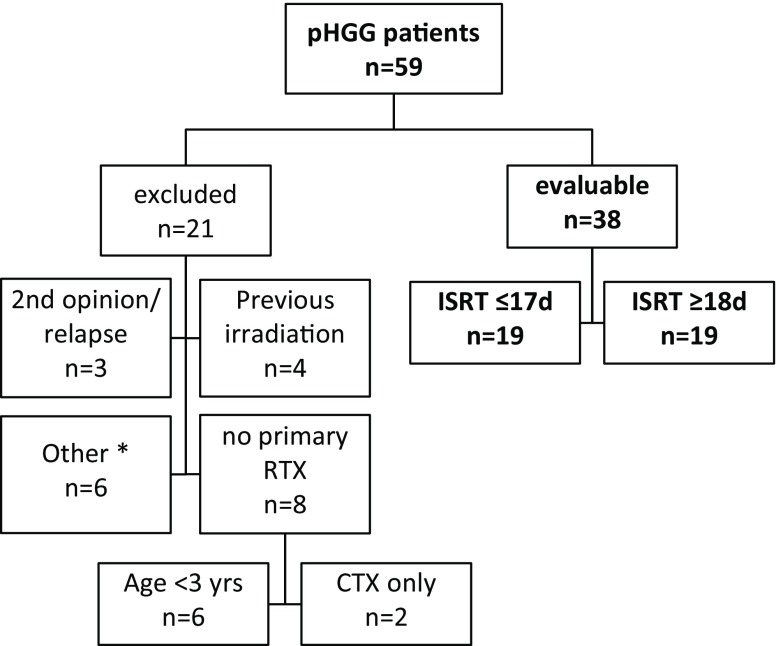
Patient evaluation. (^asterisk^Other excluded patients: *n* = 2 tumour reclassified (Glioblastoma → atypical teratoid/rhabdoid tumour; anaplastic astrocytoma → pleomorphic-xanthoastrocytoma WHO°II); *n* = 1 died before surgery; *n* = 1 RTX discontinued by choice of the parents after 2 weeks at a dose of 22.0 Gy; *n* = 2 insufficient quality of available data. *CTX* chemotherapy, *d* days, *ISRT* interval from surgery to radiotherapy, *pHGG* paediatric high grade glioma, *RTX* radiotherapy, *yrs* years)

The medical histories of 59 patients with HGG were evaluated; 38 of them met the above mentioned criteria (Fig. 1). All patients received radiochemotherapy (RCTX) following surgery. Patient characteristics are listed in Table 2. According to a median ISRT of 18 days, the cohort was divided in two groups: group 1 started simultaneous RCTX within 17 days of surgery, group 2 after ≥18 days. An exploratory analysis also evaluated the impact of an ISRT of ±28 days (see below).

**Table 2:** Patient characteristics

		Group 1 ISRT ≤17 d	Group 2 ISRT ≥18 d	Total
*Sex*	Male	9 (47.4%)	10 (52.6%)	19 (50.0%)
	Female	10 (52.6%)	9 (47.4%)	19 (50.0%)
*Age*	3–10	7 (36.8%)	12 (63.2%)	18 (47.4%)
	11–19	12 (63.2%)	7 (36.8%)	20 (52.6%)
*Grading*	WHO grade III	6 (31.6%)	11 (57.9%)	17 (44.7%)
	WHO grade IV	13 (68.4%)	8 (42.1%)	21 (55.3%)
*Location*	Hemispheric	10 (60.0%)	10 (53.3%)	20 (52.6%)
	Diencephalic	8 (33.3%)	8 (40.0%)	16 (42.1%)
	Spinal	1 (6.7%)	0 (0.0%)	1 (2.6%)
	Multifocal	0 (0.0%)	1 (6.7%)	1 (2.6%)
*Extent of resection (EOR)*	Gross total	5 (26.3%)	5 (26.3%)	10 (26.3%)
	Subtotal	4 (21.1%)	3 (15.8%)	7 (18.4%)
	Partial	2 (10.5%)	5 (26.3%)	7 (18.4%)
	Biopsy	8 (42.1%)	6 (31.6%)	14 (36.8%)
*Follow-up (median)*	Overall	62.2 months	56.6 months	60.7 months
	Range (in months)	6.9–89.2	76.6–169.4	6.9–169.4
*n (%) of total cohort*		19 (50.0%)	19 (50.0%)	38 (100%)

*CTX* chemotherapy, *HGG* high grade glioma, *ISRT* interval from surgery to radiotherapy, *RTX* radiotherapy, *WHO* World Health Organisation

### Descriptive analysis

#### Patient characteristics

The median age at diagnosis was 11.0 years (3.4–17.7); the mean age was 11.3 years (SD 4.24). Age as well as sex were evenly distributed between study groups. Histology revealed 17 WHO grade 3 and 21 grade 4 tumours, including three cases of gliomatosis. There were more patients with grade 4 tumours in group 1 than group 2, yet the difference was not statistically significant (Chi-square testing *p* = 0.10). Except for one case of a spinal glioblastoma, all tumours were located supratentorially, 7 (18.4%) bilaterally. Sixteen tumours were located in the supratentorial midline (42.1%); primary leptomeningeal dissemination was present in a single case of glioblastoma WHO grade 4.

#### Extent of resection (EOR)

A gross total resection (GTR) was achieved in 10 (26.3%), near total/subtotal resection and partial resection each in 7 patients (18.4%). Fourteen patients (36.8%) underwent a biopsy only. The number of patients with GTR was comparable in groups 1 and 2.

#### Radiotherapy

Of all patients, 84.2% (*n* = 32) underwent focal conformal cranial RTX, the patient with spinal GBM underwent focal spinal RTX and four underwent whole-brain irradiation because of gliomatosis cerebri or multifocal GBM. The single patient with primary leptomeningeal disease received craniospinal irradiation. The mean duration of RTX was 6.2 weeks. Median dose applied was 59.4 Gy (50–66.0 Gy). Two thirds of all patients (*n* = 24) received a dose ≥59.4 Gy, delivered in fractions varying from 1.6–2.0 Gy. No severe side effects were reported.

#### Adjuvant chemotherapy

All 38 patients received adjuvant systemic therapies. Details are listed in Table 3.

**Table 3:** Chemotherapy regimens applied for HGG treatment

CTX regimen	*n* =	%
*HIT-GBM-D *[37]		
MTX window	9	34.2
No window therapy	4	10.5
*PEI-based CTX incl.* *HIT-GBM-C *[36]	11	29.0
*Temozolomide, Stupp-like regimen *[38]	10	26.3
*GBM-VAX *[39]* Dendritic cell vaccine* *+* *Stupp regimen*	2	5.3
*VCR 1.5* *mg/m*^*2*^* weekly during RTX*	1	2.6
*Nimotuzumab* *+* *vinorelbin *[40, 41]	1	2.6

*MTX* Methotrexate, *PEI* Cisplatin, Etoposide, Ifosfamide, *RTX* Radiotherapy, *VCR* Vincristine

*GBM-VAX*, *HIT-GBM-D* and *HIT-GBM-C* are protocol names

#### Survival

By the end of data accrual (12/2015), 31/38 patients (81.6%) had experienced progression or disease recurrence; 30/38 (78.9%) had died. Median follow-up was 60.7 months (6.9–169.4). Median PFS was 10.4 months, with 1‑, 2‑ and 5‑year progression-free survival rates of 42.1%, 23.7% and 18.1%, respectively. Seventeen of 31 patients (54.8%) with disease progression/recurrence were switched to alternative chemotherapy protocols and 6 (19.4%) were re-irradiated, followed by alternative chemotherapy in three. In 6 patients, only supportive care was provided at the time of relapse. Documentation on relapse therapy was missing in 2 patients. Only 1/31 patients with recurrence is still alive for more than 5 years after primary diagnosis following lysate-pulsed dendritic cell (DC) vaccination at diagnosis and renewed DC vaccination after re-surgery and re-irradiation. Survivors were evenly distributed between groups (3 in group 1, 5 in group 2). Five of eight (62.5%) survivors had GTR. Median OS was 15.9 months and 1‑, 2‑ and 5‑year OS were 68.4%, 29.0% and 21.1%, respectively.

#### Interval from surgery to start of radiotherapy (ISRT)

Median ISRT was 18 days (range 5–78 days). In group 1 (receiving therapy within the first 17 days) the median interval to start of RTX was 12 days (range 5–17); in group 2 the median interval was 28 days (range 19–78). The delay of 78 days was an outlier and seen in a patient with GBM and local inconclusive biopsy who was referred late and only received treatment after re-evaluation of histology. Whereas all 4/30 patients starting RTX within the first week after surgery underwent biopsy only, ISRT was overall not significantly different in patients who only had biopsy compared to patients with debulking surgeries.

### Univariate analysis

#### Overall survival

**Fig. 2:**
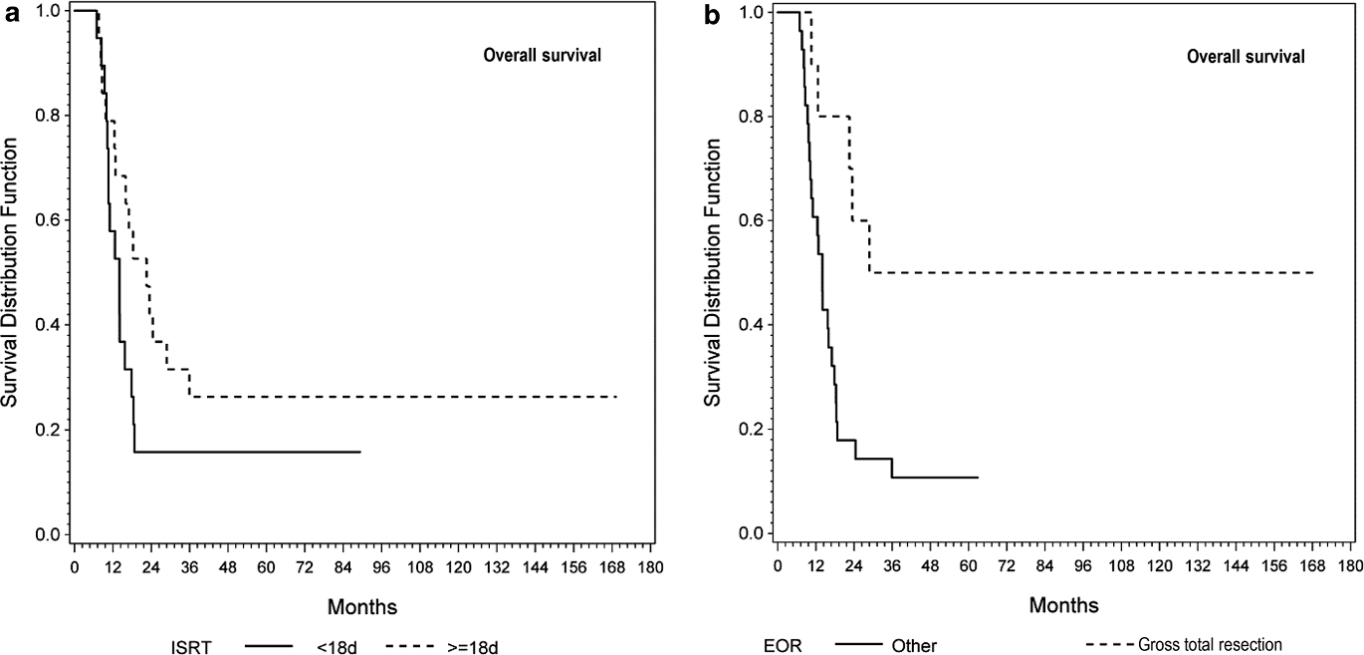
Overall survival. **a** There was no difference in overall survival between patients with an ISRT of ≤17 days vs. ≥18 days (log-rank: *p* = 0.143). **b** EOR: patients with gross total resection achieved a significantly superior overall survival when compared to all other patterns of resection (*p* = 0.007). (*EOR* extent of resection; *ISRT* interval from surgery to radiotherapy)

There was no statistically significant difference in overall survival (OS) between groups (log-rank: *p* = 0.143) (see Fig. 2a). Tumour grade (improved OS for grade 3 tumours, *p* = 0.020) and EOR significantly influenced OS (*p* = 0.022). When comparing GTR to all other patterns of resection, significance was increased (*p* = 0.007) (see Fig. 2b). Resulting from univariate Cox regression analysis the hazard ratio (HR) of ISRT (continuous variable, log2-transformed) as a prognostic factor on OS was not statistically significant (HR 0.78, 95% confidence interval [CI] 0.51–1.18, *p* = 0.231). Grade (HR 2.33, CI 1.13–5.05, *p* = 0.022) and EOR (HR 0.62, CI 0.44–0.84, *p* = 0.002) statistically significantly influenced OS. OS was improved even further by GTR when compared to all other resections combined (HR 0.30, CI 0.11–0.72, *p* = 0.006). Age was not identified as a statistically significant risk factor (*p* = 0.292).

#### Progression-free survival

PFS was not statistically significantly different between groups 1 and 2 (log-rank: *p* = 0.126). Grade (*p* = 0.020) and GTR (*p* = 0.021) were statistically significantly associated with a superior PFS. In the univariate Cox regression models ISRT as a continuous variable (log2-transformed; HR 0.74, CI 0.50–1.11, *p* = 0.142) and dichotomised by group 1 and 2 (comparing longer to shorter intervals: HR 0.58, CI 0.28–1.17, *p* = 0.130) as well as age (HR 0.97, CI 0.90–1.04, *p* = 0.361) were not statistically significant; grade (HR 2.31, CI 1.13–4.98, *p* = 0.022) and EOR (HR 0.65, CI 0.47–0.87, *p* = 0.004) were statistically significantly associated with PFS.

### Multivariable analysis

#### Overall survival

EOR remained statistically significant in multivariable analysis (HR 0.23, CI 0.07–0.57, *p* = 0.0009) and grade also reached significance (HR 3.02, CI 1.26–7.81, *p* = 0.013), whereas an ISRT of ≥18 vs. ≤17 days did not (HR 0.82, CI 0.35–1.91, *p* = 0.643). Considering ISRT as a continuous variable did not alter this finding (HR 0.95, CI 0.63–1.46, *p* = 0.821).

#### Progression-free survival

Grade (HR 2.76, CI 1.21–6.69, *p* = 0.015) and EOR (HR 0.28, CI 0.10–0.66, *p* = 0.003) were statistically significantly associated with PFS in multivariable analysis, but not ISRT dichotomised by group 1 and 2 (HR 0.71, CI 0.31–1.55, *p* = 0.390) or as a continuous variable (HR 0.85, CI 0.58–1.28, *p* = 0.432).

### Additional analyses

In order to investigate the influence of adjuvant chemotherapy, we performed a survival analysis comparing patients receiving TMZ (10/38) to the remaining patients. Patients treated with TMZ exhibited a significantly better OS (*p* = 0.043) and PFS (*p* = 0.045) in univariate analysis. The survival benefit in OS was also found in multivariate analysis (*p* = 0.020, HR 0.35, CI 0.12–0.86), but showed only a trend for PFS (*p* = 0.098, HR 0.48, CI 0.17–1.14). Data on O-6-methylguanine-DNA methyltransferase (MGMT) status were not available.

When exploring a possible difference between patients treated within four weeks after surgery (*n* = 29) to those treated thereafter (*n* = 9), no significant difference either in OS (*p* = 0.655) or PFS (*p* = 0.447) could be identified.

## Discussion

Like its adult counterpart paediatric high grade glioma (pHGG) has an unfavourable prognosis. In order to improve survival it is important to recognise potential influencing factors. Molecular subgrouping of pHGG [24] may influence treatment decisions in the future (e. g. presence of BRAF V600E mutations [25] or of a hypermutated phenotype [26] for which specific therapies are available). Since molecular subgrouping is often feasible in real time before starting adjuvant therapy [18], it is fundamental to identify a time limit that is safe to perform all necessary molecular testing. Prompted by a recent study involving a targeted drug that required a delay in start of radiotherapy for 4 weeks [27] we retrospectively analysed our consecutive series of patients above 3 years of age with HGG. As data on the best time point of radiotherapy in children with HGG are lacking, the present analysis evaluated a possible influence of the interval from surgery to radiotherapy (ISRT) in this patient population. Unlike those postulated from other tumour entities [2] and defying radiobiological theorems [1], numerous adult studies could not detect a presumed negative influence of ISRT on patient survival in HGG [8–18]. A large study conducted by Blumenthal et al. [13] even suggested a favourable influence of prolonged waiting times until start of RTX. Similar to this observation ISRT could not be identified as a significant risk factor in our single-centre population of pHGG. Multiple factors may possibly influence such a finding like an uneven distribution of age, tumour grade or EOR. Whereas indeed our cohort of patients with the shortest ISRT comprised more patients with grade IV tumours, there was no significant difference in EOR or age. Studies involving adults found similar and even further bias: patients with unfavourable baseline characteristics and highly aggressive tumours seem to be over-represented in cohorts with shorter ISRT [6, 13–16]. In the large study by Blumenthal et al. [13] the cohort with shortest delays had worse scores of performance status and fewer gross total resections. This is underlined by findings of Spratt et al. [6] and Wang et al. [16]. Lai et al. [14] demonstrated that patients with biopsy only were more likely to be irradiated sooner after surgery. In an analysis by Wehming et al. [15] patients with an ISRT <24 days suffered more often from a grade IV tumour, had a minor EOR and a higher median age. Irwin et al. described a direct correlation of a better performance status to a prolonged ISRT [3]. Clinicians thus tend to apply RTX early in patients with a large tumour load and poor prognosis. This bias may consequently mask the effects of late treatment initiation.

In the present analysis only nine of 38 children were treated with an ISRT of more than 28 days. The overall length of ISRT in our cohort was therefore shorter than in the published data in adult HGG [13, 15, 18]. But, while overall ISRT seems longer in the adult population, only few patients with HGG had very long delays above 6 weeks, which is in contrast to studies in breast or head and neck cancer that identified a detrimental effect of delayed RTX [2]. In cohorts including patients with very long delays survival was impaired and it was deduced that delaying RTX beyond 6 weeks after surgery was a negative predictive factor [4–7]. Han et al. [17] even postulated an optimal time window of 30–34 days post-surgery, after which survival worsened. In contrast, studies postulating a positive effect of postponing RTX had a minority of patients with prolonged waiting times over 6 weeks, e. g. 0.7% [13] and 2.4% [14], respectively. Considering that in our study 29/38 patients started irradiation within 28 days after surgery this 4‑week interval may be too narrow to cause significant differences in outcome. Yet, when comparing patients receiving RTX within four weeks or thereafter we could not even identify a trend towards a significant difference.

In addition to ISRT we also analysed other factors possibly influencing outcome in pHGG. Previous findings identified a higher extent of resection (EOR) [28–31] and lower tumour grade [32, 33] as major positive predictive factors for improved survival in pHGG. In accordance with the literature it was possible to reproduce the favourable influence of a greater EOR in univariate as well as multivariable analysis. Presence of a higher tumour grade could also be established to be associated with poorer PFS and OS. Young age as a predictor for favourable outcome [30, 32, 34] could not be evaluated in our cohort since patients under the age of 3 years (i. e. not receiving radiotherapy [19]) were excluded from the study.

Radiotherapy following surgery is considered the gold standard of HGG therapy in patients older than three years of age. Adjuvant chemotherapy was also used in all our patients but regimens varied over the past 2 decades. Interestingly and in contrast to previous findings [35], temozolomide (TMZ) seemed to be superior when compared to all other therapies in an exploratory analysis (the MGMT status being unknown in our retrospective cohort).

## Conclusion

Our data therefore suggest the absence of a significant survival cost of moderate delays of simultaneous RCTX and waiting times of up to four weeks after surgery to be safely tolerable. The four week time frame may be used for molecular analysis needed for including patients with pHGG in future prospective clinical trials.
